# Exploring the Effect of Team-Environment Fit in the Relationship Between Team Personality, Job Satisfaction, and Performance

**DOI:** 10.3389/fpubh.2022.897482

**Published:** 2022-06-20

**Authors:** Xin Lin, Ornlatcha Sivarak, Tung-Hsiang Chou, Yu-Ting Lin, Untung Rahardja, Athapol Ruangkanjanases, Yu-Chun Lin, Shih-Chih Chen

**Affiliations:** ^1^School of Economics and Management, Northeast Electric Power University, Jilin, China; ^2^Mahidol University International College, Mahidol University, Nakhon Pathom, Thailand; ^3^Department of Information Management, National Kaohsiung University of Science and Technology, Kaohsiung, Taiwan; ^4^Department of Information Management, Tatung University, Taipei, Taiwan; ^5^Science and Technology Faculty, University of Raharja, Tangerang, Indonesia; ^6^Chulalongkorn Business School, Chulalongkorn University, Bangkok, Thailand

**Keywords:** team personality, team-organization fit (T-O fit), team-job fit (T-J fit), team job satisfaction, team performance

## Abstract

This study explores whether team-organization fit (T-O fit) and team-job fit (T-J fit) play a mediating role between team personality, team job satisfaction, and team performance. Conscientiousness and openness to experience are common antecedents of team personality. Additionally, T-O fit and T-J fit are derived from person-environment fit theory, which is used to discuss the interaction between team members and the environment that affects behavior. The research purpose is to understand the factors that affect job satisfaction and performance from a team perspective. This is different from previous studies based on an individual perspective. The research object of this study has 365 respondents from 81 teams in different industries, and the structural equation modeling is applied to the empirical analysis. The research results show that T-J fit has a significant mediating effect on team personality and team job satisfaction. The team job satisfaction has also a significant mediating effect on team personality and team performance. Therefore, when team members recognize their work, they work harder to achieve team job satisfaction and performance. This study suggests that companies not only pay attention to the work abilities of employees, but also understand the fit between them and their jobs.

## Introduction

With the advent of economic globalization and knowledge-based economy, many new topics have been put forward for the research about organizational behavior. Furthermore, as the change of politics, economy, and international situation, organizations encounter many internal changes, such as strategy, structure adjustment, system innovation. The complexity of organizational functions and tasks is increasing. It is only by replacing individuals with teams as the basic unit of organization, bringing together the capabilities and characteristics of individuals, leveraging the power of the team, and providing better responsiveness, task-oriented effort, and organizational productivity, that the key to achieving corporate vision and goals are achieved (1).

In the past, traditional recruitment often focused on finding a person according to job responsibilities and qualifications required by job functions (e.g., person-job fit). It was usually assumed that as long as people had sufficient professional knowledge, skills and abilities, they would be able to perform the tasks, duties, and responsibilities of the job. This kind of recruitment based on person-job fit does not consider whether personality traits and values of employees are compatible with organizational culture and philosophy. Employees may resign because they feel that they are not suitable for the company. If turnover occurs frequently, not only will the organization take more time and cost to recruit, but the morale of employees will also be low. This phenomenon is not conducive to organizational development. Therefore, when the organization recruits new employees or selects members of the team, in addition to considering the professional skills of the candidates, it should also consider whether their personality traits are consistent with the organizational culture or team personality. Obviously, the traditional human resource management system built based on person-job fit cannot meet the needs of organizational development (2).

The issue about the compatibility of personality traits with various occupations have been emphasized, as is the issue regarding person-environment fit [e.g., (3, 4)]. This study argues that individual factors (e.g., technology and values) and organizational factors (e.g., working conditions, organizational culture, and organizational climate) can be considered simultaneously, the research results are more objective and accurate. Moreover, the research field of personal and organizational fit is most often discussed (e.g., person-organization fit), for example the interaction between individual behavior and organizational behavior, person-organization fit in the employment process (e.g., selection, employment decision, and career choice decision). The person-environment fit (especially, person-organization fit) has been continuously discussed [e.g., (2, 5–8)]. Thus, the research on person-organization fit has broaden new horizons for the development of organization behavior and human resource management. On the other hand, personality is also an important factor that affects team functioning and performance (9). However, most research has discussed the individual-level personality. Subsequently, scholars have applied the Big Five personality traits to explore the impact of team-level (group-level) personality on performance. They have argued that the Big Five personality traits can indeed be used to deal with team-level personality, and to verify that team personality (especially conscientiousness and openness to experience) can affect team performance [e.g., (9–11)].

In the past literature, the consistency and fit between the individual and the environment have attracted the attention of researchers (12). As a result, the person-environment fit (P-E fit) theory has been developed, which emphasizes the state of individual and environmental fit. Since the late 1980s, scholars have discussed P-E fit. Subsequently, there was much research on the relationship between P-E fit and behavior and outcomes of work. For example, Kristof-Brown et al. (4) indicate that P-E fit can predict and explain multiple behaviors and attitudes, such as organizational commitment, organizational citizenship behavior, turnover intention. The relationship between job satisfaction and performance has been paid attention to by researchers [e.g., (13–15)]. However, there are relatively few researches on team-job fit (T-J fit) and team-organization fit (T-O fit). A few researches have tested multiple types of fit simultaneously (16). There is no large number of empirical results showing how T-O fit and T-J fit affect team job satisfaction and team performance.

According to the above perspective, this study explores the extension of P-E fit theory from the team-level. Thus, this research purpose is to empirically demonstrate the mediating effect of T-O fit and T-J fit between team personality, team job satisfaction and team performance. Structural equation modeling was used as the analytical method. The next sections include compiling relevant literature and research findings, proposing research hypotheses and models, and then conducting empirical analysis and discussing the findings. It is hoped that the research findings will fill the gaps in the relevant research fields and serve as a reference for companies to develop strategies to recruit and select team members and to promote team job satisfaction and team performance.

## Background and Literature Review

### Team Personality

This study mainly takes the team as the research and analysis unit. Scholars have defined the team [e.g., (17–20)]. This study refers to the views of scholars and defines a team as a group of people with sufficient skills who are willing to commit to each other to achieve a common goal and be responsible to each other in the process.

In the field of human resource management, personality traits have been discussed. Some scholars are also concerned about the team-level personality [e.g., (9, 10, 21)]. “Personality traits” are considered to be an individual's stable and unchanging psychological characteristics, and are often used to explain or predict a person's behavior. “Team personality” is considered to be a collection of personality traits of members that influence the process and results of team development. Hoch and Dülleborn (21) proposed that team personality is a deep-level aspect, because it is the integration of team members' psychological characteristics and affects team process and results.

Neuman et al. (9) advocated that team personality refers to the average of personality traits of team members and the differences among them. In addition, team personality can be described as the aggregation and configuration of personality traits in a team, and they affect the development and outcome of the team (10). A few researches on team personality have mainly been conducted adopting Big Five personality traits [e.g., (11, 22)]. Therefore, this study also applies Big Five personality traits to measure the team personality.

#### Individual-Level and Team-Level Personality Traits

The Big Five personality traits are some stable and long-term specific responses of individuals. Compared with emotion or state, personality traits are relatively unchanged. The Big Five personality includes neuroticism, agreeableness, extroversion, conscientiousness, and openness to experience. It is used to explain the differences in individual personality traits (23). It is one of the important measurements in modern psychology. The five personality traits of the individual remain stable over time. Each personality trait makes an individual inclined to certain behaviors. In a team, the personality traits (such as conscientiousness) possessed by team members are gathered to form team personality in each dimension (24). In addition, in terms of team development, some researches have suggested that personality traits (e.g., conscientiousness and openness to experience) have a positive impact on team operations. Their research found that team personality is the main predictor of team performance (21, 25).

#### Reasons for Conscientiousness and Openness to Experience as the Research Focus

LePine et al. (26) advocate that openness to experience is a good predictor of individual innovativeness. On the other hand, Peeters et al. (22) had comprehensively analyzed the team personality and proposed that conscientiousness positively affects team performance. They also verified that the personality trait “conscientiousness” can predict both individual performance and group performance. In addition, conscientiousness and openness to experience are mostly valued in organizational change literature. However, there is a significant difference between individual-level and team-level personality traits on teams (27). Thus, referring the viewpoints of previous research, conscientiousness and openness to experience were the focus of this study.

### Team-Environment Fit

Which one of individual or environmental characteristics has a greater impact on behavior and job outcomes is an important issue for the human resources department. Lewin (28) proposed “fit” based on interactionist theory and emphasized that the interaction between the individual and the environment influences behavior. Then the personal-environmental fit (P-E fit) theory was developed. Jansen and Kristof-Brown (29) classified P-E fit into five categories, including person-vocation fit (P-V fit), person-organization fit (P-O fit), person-job fit (P-J fit), person-group fit (P-G fit), and person-person fit (P-P fit). Among them, P-O fit, and P-J fit were most discussed. Many scholars have adopted P-O fit and P-J fit as the main independent variables to explore the impact on behaviors, attitudes, and work results such as job satisfaction and job performance [e.g., (30, 31)]. Until now, the P-E fit has continued to be discussed. This is because scholars are convinced of the existence of the P-E fit. Furthermore, some scholars have further pointed out that P-E fit does not only static “exist” but also changes with time. Therefore, they advocate that when discussing issues regarding the P-E fit theory, in addition to integrating other theories or factors, “time” should also be considered (32). Moreover, some researchers are interested in team-level issues. Team-environment fit (T-E fit), including team-organization fit (T-O fit) and team-job fit (T-J fit), has also been considered [e.g., (3, 33–35)]. Compared to the P-E fit, there are very few papers on the T-E fit. Hence, this study attempts to empirically demonstrate the impacts of T-O fit and T-J fit on team job satisfaction and team performance at the team level.

#### Team-Organization Fit

More and more people realize that employees are an important resource, which makes researchers continue to be interested in the impact of P-O fit on personal work attitude and satisfaction (36). According to the opinions of many scholars, P-O fit is defined as the similarity of values between individuals and organizations and should be used as an important evaluation when the organization recruits and selects employees [e.g., (2, 5–8)]. Lam et al. (37) suggested that a person may be attracted by organizations with similar characteristics. For example, a gregarious person may look forward to working in an organization that emphasizes teamwork. If employees perceive to fit into the organization, they feel that they are part of the organization (38). Therefore, P-O fit is an important condition for the team to select members (39). On the other hand, scholars have different interpretations of T-O fit. Researches have defined T-O fit as a fit between team and organizational values (3, 35). Sekiguchi (33) pointed out that the concept of T-O fit is derived from the Attraction-Selection-Attrition (ASA) model. The ASA model emphasizes that individual and organizational characteristics should be similar. In other words, the team and organizational characteristics should also be similar.

#### Team-Job Fit

Caldwell and O'Reilly (40) defined P-J fit as the consistency of personality traits with the workplace, or the compatibility of an individual with a specific job. In other words, the skills of employees must meet job requirements. That is, it emphasizes the fit of the individual's personality traits and abilities with the job or task. Scholars have found that P-J fit affects work behavior and outcome (e.g., job satisfaction, job performance, turnover intention, and organizational identification) (41, 42). Later, some scholars also paid attention to T-J fit. Ellis et al. (34) suggested that T-J fit can be measured by the correlation between team personality and job requirements.

### Team Job Satisfaction

In addition to individual job satisfaction, team job satisfaction has also received attention from researchers [e.g., (43)]. Team job satisfaction refers to the feelings or emotions of team members about job and the workplace (44). Team members with higher job satisfaction may have a positive attitude toward his job (45). On the other hand, Downes et al. (46) found that team personality is positively correlated with P-O fit, and indirectly affects goal achievement and job satisfaction. Researches have also shown that T-O fit is an important factor affecting job satisfaction (1, 31). Each individual's feelings of satisfaction are different. However, the mainstream value of the individual may be consistent with the value of the organization. The more an individual's values fit the organization's value, the higher the individual's satisfaction with the organization.

### Team Performance

Team performance not only reflects the overall strength of a team and the group's contribution to its enterprise but also reflects the efforts of each member of the team. Some scholars have proposed that team performance refers to the extension that team members jointly achieve mission and goals (25). Team members must participate in the team process/teamwork to achieve organizational tasks and goals through interrelated attitudes, cognitions, and behaviors (25, 47). Teamwork is a dynamic process. Team performance is one of the most important methods of evaluating teamwork (48). Since team performance is the result of interactions among members or between them and the environment, many researches have discussed the factors that influence team performance, such as team personality, P-E fits, and job satisfaction (10, 25, 49–52).

According to the above literature reviews, this study summarizes and defines each variable (see Table 1).

**Table 1:** Operational definitions.

**Dimensions**	**Variables**	**Descriptions**	**References**
Team personality (TP)	Conscientiousness	It means that the behavior of conscientious team members involved in achieving goals and solving problems.	(25, 53)
	Openness to experience	It means that the adaptability and responsiveness of team members in a dynamic team environment.	
T-O fit (TO)		It means that the individual and the organization have the same values.	(54, 55)
T-J fit (TJ)		It means that the supply of jobs meets the needs of the employees or that the employees' abilities meet the requirements of the job.	(56, 57)
Team job satisfaction (SA)		It means that workers' feelings, attitudes, and affective responses to work, experiences, and the workplace.	(44, 58)
Team performance (PER)		It means that the results and goals that team members achieve after mutual dependence and interaction.	(25, 47)

## Research Methods

### Hypothesis and Model

Based on the research purpose and through the literature review, this subsection explores the relationship between team personality, T-O fit, T-J fit, team job satisfaction and team performance, proposes hypotheses, and constructs a research model.

#### Team Personality and T-O Fit, T-J Fit

The current environment is changing rapidly, and organizations must adapt to such an environment in order to develop sustainably. Kim et al. (59) believe that employee enthusiasm can moderate the relationship between the organization's socialization strategy and P-O fit. Members with openness to experience are committed to fit the team (60), and team execution and responsiveness are enhanced (61). Thus, when a team has openness to experience, it fit the environment more actively. On the other hand, research has confirmed that a high degree of conscientiousness is the most effective predictor of team performance, which helps members focus on completing team tasks, team development and performance improvement (62). Generally speaking, in a team, a member with conscientiousness is more likely to become the task leader. A responsible team should create an environment that encourages and rewards members' responsibility, so as to motivate responsible members to show greater enthusiasm (21). In addition, a high degree of team responsibility leads to team members willing to cooperate and participate in team tasks. A high level of team responsibility also helps improve team performance. Based on the above literature review, this study infers that team personality is related to both T-O fit and T-J fit, so the following hypothesis is proposed.

*H1a: Team personality has a positive relationship with T-O fit*.
*H1b: Team personality has a positive relationship with T-J fit*.

#### T-O Fit, T-J Fit, Team Job Satisfaction, and Team Performance

Generally speaking, job satisfaction is considered a psychological characteristic of a person, and this psychological characteristic is reflected in his work. In addition, when employees' skills and abilities are in line with their job content, their performance and satisfaction will be improved. This indicates that the perception of job satisfaction is the result of the interaction between the person and the work environment (63, 64). Brkich et al. (42) proposed that employees feel more organizational identity when they believe that their values are consistent with the values of the organization and verified a significant relationship between individual and job fit and employees' job satisfaction. Moreover, Xiao et al. (65) explored the impact of P-E fit on the job satisfaction of medical workers. They found that P-E fit (including P-J fit and P-G fit) has a significant positive impact on job satisfaction. There is a research examining the relationship between police officers and their work environment. The results show that when police officers have highly aligned with the overall goals and direction of the organization, they also have high job satisfaction (66). This study extends the above-mentioned arguments and research findings, and inferences that both T-O fit and T-J fit have an impact on team work satisfaction.

On the other hand, performance reflects the degree of an individual's job responsibilities and organizational goals completed in a period. It is an important behavioral outcome variable of the fit between individuals and organizations. When there is a certain degree of fit between the characteristics of individuals and organizations, the performance is higher. Amarneh and Muthuveloo (52) confirmed that there was a positive correlation between individual fit to job and behavior outcome variables, such as job satisfaction, low work stress, performance, attendance rate, and retention rate. In addition, in temporary organizations, P-E fit (including P-O fit, P-G fit, and P-J fit) has a significant impact on task performance and innovation performance (67). Lim et al. (2) and Dhir and Dutta (6) demonstrated that both P-O fit and P-J fit are positively and significantly related to job satisfaction. Some scholars have proposed that the relationship between leaders can be regarded as the relationship between the team and the organization in a hospital. They also argued that the better the relationship between the teams or the higher the trust between the teams and the organization, the better the team performance (68). Most researches on fit tend to have positive effects, but some researches point out that high fit has some negative effects, which affect the adaptability and innovation ability of the organization (34). However, most of the researches on P-O fit focuses on individual performance. This study argues the higher fit brings more benefits from the team level.

In general, organizational performance is achieved when employees are satisfied with their work. A large number of researches indicated that job satisfaction has a positive impact on performance [e.g., (49–51, 69)]. Furthermore, Khadivi et al. (70) emphasized that job satisfaction is related to organizational performance. Thus, this study infers that team job satisfaction is also related to team performance. According to the previous research, this study establishes the following hypotheses.

*H2a: T-O fit has a positive relationship with team job satisfaction*.
*H2b: T-J fit has a positive relationship with team job satisfaction*.
*H3a: T-O fit has a positive relationship with team performance*.
*H3b: T-J fit has a positive relationship with team performance*.
*H4a: Team job satisfaction has a positive relationship with team performance*.

#### The Mediating Effect of Team-Environment Fit

As mentioned above, team personality is the average of the personality traits of team members (9). O'Neill and Allen (11) found team personality significantly affect team performance. In addition, Sortheix et al. (71) advocate T-E fit refers to the compatibility and consistency of team characteristics and workplace perceived by team members. Most employees expect that the team they will participate in has the characteristics of T-E fit. T-E fit is a psychological resource (72). In addition, the team can adapt to the environment, which helps members integrate into their work, thereby increasing personal professional satisfaction (73). In addition, Ellis et al. (34) suggested that T-J fit can be measured by the correlation between team personality and job requirements. However, the T-E fit needs further discussion and verification (74, 75). As a result, research has explored individual-environment (organizational and job) fit and found that individual-environment fit is related to job satisfaction and job performance (42, 63, 64). Finally, job satisfaction is positively related to organizational performance; job satisfaction is also affected by some factors (such as supervisor, team, and organization) (70). Based on the literature reviews, this study infers that T-O fit and T-J fit have a mediating effect between team personality, team job satisfaction, and team performance; team job satisfaction has a mediating effect between T-J fit and team performance. Then, the following hypotheses are proposed.

*H5a: T-O fit has a mediating effect between team personality and team job satisfaction*.
*H5b: T-O fit has a mediating effect between team personality and team performance*.
*H6a: T-J fit has a mediating effect between team personality and team job satisfaction*.
*H6b: T-J fit has a mediating effect between team personality and team performance*.
*H7a: Team job satisfaction has a mediating effect between T-J fit and team performance*.

**Figure 1:**
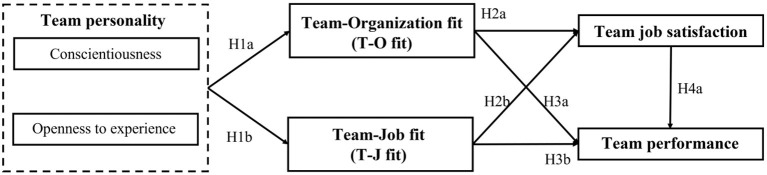
Research model.

According to the above discussion and hypotheses, the following research framework is proposed in Figure 1.

### Research Process and Method

Among the relevant measurement tools, many researchers have developed five personality traits, for example, Goldberg (76) and Saucier (77). This study adopts the International English version of Big Five Mini markers (International English Big Five Mini markers), which developed by Thompson (78). Next, this study refers to the research of Cable and DeRue (57) to develop the scale about T-O fit and T-J fit and adopts the scale regarding job satisfaction developed by Brayfield and Rothe (79). Finally, the Barrick and Stewart's (80) scale was adopted to measure team performance. This study used the Likert scale.

This study selects the teams within some enterprise as the research object. Mainly for the team of 3–5 people, a total of 100 sets of 500 questionnaires were sent out. Through the questionnaire survey, the team members were directly measured, and 395 were recovered, with a recovery rate of 79%. After the index screening, 365 sets were obtained, 30 of which were eliminated in this survey, and the effective questionnaire recovery rate was 73%. The reasons for elimination are incomplete answers and multiple answers to one question. At the team level, 85 team data were collected, 4 teams have <60% effective samples that are not included, so there are 81 effective samples.

SPSS 21 and AMOS 24 are adopted as the analysis tools. Scholars have suggested that the Structural Equation Model (SEM) is suitable for investigating the effects between the various structures and verifying the suitability of the research model (81, 82). Additionally, the two-stage SEM validation procedure is to examine the suitability of the measured modes before the structural modes are examined (83, 84). Consequently, this study utilizes SEM to perform inferential statistics. Furthermore, this study followed the suggestion of some scholars to use Bootstrapping for the examination procedure of mediation effects [e.g., (85–87)] and repeated the sampling 5,000 times.

## Research Results

### Descriptive Statistics Analysis

The descriptive statistics of individual-level data (*n* = 365) are described in Table 2. The respondents are more female (60.00%) than male (40.00%), and more single (73.70%) than married (26.30%). The respondents under the age of 25 are the most, accounting for 31.00%. In terms of education level, the majority of respondents are college/university degrees, accounting for 75.34%. Respondents with 1–3 years of working experience are the most (32.60%). Respondents are mainly distributed in private enterprises (89.04%). The respondents in the information department are the most, accounting for 24.93%. At the unit level, 48.77% of the respondents work at the department level. Respondents are the most in the financial services industry, accounting for 23.29%.

**Table 2:** Distribution of individual-level data (*n* = 365).

**Items**	**Frequency**	**Percent**
**Gender**		
Female	219	60.00%
Male	146	40.00%
**Marital status**		
Single	269	73.70%
Married	96	26.30%
**Age**		
25 or below	113	31.00%
26–30	108	29.59%
31–35	61	16.71%
36–40	44	12.05%
41–45	17	4.66%
46 or above	22	6.03%
**Education**		
High school or below	22	6.03%
College/University	275	75.34%
Master's degree	61	16.71%
Doctor's degree	7	1.92%
**Job tenure**		
<1 year	90	24.66%
1–3 years	119	32.60%
4–6 years	65	17.81%
7–9 years	37	10.14%
More than 10 years	54	14.79%
**Company nature**		
Government agencies	32	8.77%
State-owned enterprises	8	2.19%
Private enterprises	325	89.04%
**Department nature**		
R&D	30	8.22%
Quality control	14	3.84%
Customer services	45	12.33%
Marketing/sales	55	15.07%
Planning	14	3.84%
Administration	55	15.07%
Information	91	24.93%
Purchasing	5	1.37%
Human resources	5	1.37%
Production/manufacturing	3	0.82%
Accounting/cashier	45	12.33%
Others	3	0.82%
**Unit level**		
Section	33	9.04%
Subsection	13	3.56%
Division	82	22.47%
Department	178	48.77%
Others	59	16.16%
**Industry**		
Financial service	85	23.29%
Traditional manufacturing	20	5.48%
Communication services	33	9.04%
High-tech manufacturing	32	8.77%
Information service industry	79	21.64%
Medical services	20	5.48%
Retail	40	10.96%
Real estate	9	2.47%
Others	47	12.88%

Next, this study uses Table 3 to summarize the team-level data (*N* = 81). The interviewed teams are mainly distributed in private enterprises (88.89%). The team in the information department is the largest, accounting for 25.93%. In terms of unit level, 49.38% of teams belong to departments. The surveyed teams are the largest in the information service industry, accounting for 23.46%.

**Table 3:** Distribution of team-level data (*N* = 81).

**Items**	**Frequency**	**Percent**
**Company nature**		
Government agencies	7	8.64%
State-owned enterprises	2	2.47%
Private enterprises	72	88.89%
**Department nature**		
R&D	7	8.64%
Quality control	3	3.70%
Customer services	10	12.35%
Marketing/sales	11	13.58%
Planning	3	3.70%
Administration	12	14.81%
Information	21	25.93%
Purchasing	1	1.23%
Human resources	1	1.23%
Production/manufacturing	1	1.23%
Accounting/cashier	10	12.35%
Others	1	1.23%
**Unit level**		
Section	7	8.64%
Subsection	2	2.47%
Division	19	23.46%
Department	40	49.38%
Others	13	16.05%
**Industry**		
Financial service	18	22.22%
Traditional manufacturing	5	6.17%
Communication services	6	7.41%
High-tech manufacturing	7	8.64%
Information service industry	19	23.46%
Medical services	5	6.17%
Retail	8	9.88%
Real estate	2	2.47%
Others	11	13.58%

Since the variables in this study are all at the team level, and the measurement data recovered are at the individual level, it is necessary to integrate individual-level data into the team level before statistical analysis. According to the *r*_*wg*(*j*)_ index proposed by James et al. (88), the data integration of each variable is tested, and the calculation program is compiled under SPSS 21 to calculate the internal consistency of each dimension. When *r*_*wg*(*j*)_ is higher than 0.7, there is a high intragroup consistency, which indicates that it is reasonable to add the data from each team member to the team level. Table 4 shows that the proportion of *r*_*wg*(*j*)_ index of each variable above 0.7 is above 80%, and the average value of *r*_*wg*(*j*)_ index of each variable is above 0.8 except for team performance 0.795, which is very close to 0.8. The intragroup consistency of all variables was high. Therefore, the data can be integrated at the individual level and converted into team-level data for analysis, that is, the average number of all individuals in each team is used as the score of the team on a certain variable.

**Table 4:** Within-group interrater reliability—rwg(j) (*N* = 81).

**Variables**	**rwg_(*j*)_**
Conscientiousness	0.884
Openness to experience	0.869
T-O fit	0.804
T-J fit	0.811
Team job satisfaction	0.817
Team performance	0.795

### Reliability and Validity Analysis

First, this study adopts Cronbach's α to measure the stability of the questionnaire. Peterson (89) thinks that the Cronbach's α of general total scale is better than 0.80, and the Cronbach's α of subscale is better than 0.70, If the Cronbach's α of the total scale is <0.80 and the Cronbach's α of the subscale is <0.60, the items should be revised or deleted. The reliability of each variable shows in Table 5. All Cronbach's α are >0.8, indicating that the questionnaire is reliable.

**Table 5:** Reliability and validity.

**Dimensions**	**Variables**	**Items**	**Cronbach's α**	**Factor loadings**	**CR**	**AVE**
TP	Conscientiousness	8	0.862	0.509–0.826	0.850	0.549
	Openness to experience	8	0.862			
TO	Value	3	0.903	0.872–0.948	0.909	0.593
TJ	Primary demand	3	0.906	0.560–0.858	0.874	0.354
	Self-actualization	3	0.911			
	Self-esteem	4	0.924			
	Capacity	4	0.855			
	Job requirement	4	0.911			
SA		3	0.864	0.955–0.958	0.947	0.734
PER		8	0.947	0.782–0.920	0.939	0.436

*TP, team personality; TO, T-O fit; TJ, T-J fit; SA, team job satisfaction; PER, team performance; CR, composite reliability; AVE, average variance extracted*.

Second, this study used confirmatory factor analysis (CFA) to analyze the construct validity of each scale. Before confirmatory factor analysis (CFA), this study used the item pooling method to reduce the items and used the aggregate score as the observation index to reduce the error and irrelevant variation and to reduce the stability of the observed variables and reduce the possibility of error increase caused by estimation parameter inflation. In addition, T-J fit scale of this study has 18 items in total, which are divided into five items after the projected merger. Scholars have suggested that the internal consistency reliability of each scale should be tested after the merger. This study found that the reliability increased slightly, which exceeded the standard value, indicating that the next step of analysis can be carried out.

In this study, the convergent validity of the study is examined by average variance extracted (AVE). The AVE is the average explanatory variation of each dominant variable of a potential variable to the potential variant. The AVE of each dimension must be >0.5 (90). The composite reliability (CR) of the five dimensions is between 0.850 and 0.947, which shows that the internal consistency of the potential dimension is high. The AVE is between 0.595 and 0.857, indicating that the potential dimension has a high reliability and convergence ability. Then, the factor loadings for all the dimensions are greater than the value of 0.5 suggested by Hair et al. (91), indicating that the questions for these dimensions are consistent with the convergent validity. The results of the tests of convergent validity are presented in Table 5.

Torkzadeh et al. (92) proposed that the discriminative validity of the measurement can be used to calculate the confidence interval of the correlation coefficient between the dimensions using Bootstraping. If the confidence interval does not contain 1, it means that it has discriminative validity. Table 6 shows that the confidence intervals of the correlation coefficients between the dimensions do not contain 1, indicating that the measurement has discriminative validity.

**Table 6:** Discriminant validity (*N* = 81).

**Dimensions**	**Correlation coefficients**	**Confidence intervals (90%)**
(TP, TO)	0.172	(−0.014, 0.372)
(TP, TJ)	0.317	(0.107, 0.508)
(TP, SA)	0.266	(0.074, 0.453)
(TP, PER)	0.368	(0.146, 0.536)
(TO, TJ)	0.637	(0.453, 0.756)
(TO, SA)	0.632	(0.461, 0.746)
(TO, PER)	0.676	(0.550, 0.769)
(TJ, SA)	0.839	(0.710, 0.927)
(TJ, PER)	0.779	(0.686, 0.844)
(SA, PER)	0.798	(0.708, 0.861)

*TP, team personality; TO, T-O fit; TJ, T-J fit; SA, team job satisfaction; PER, team performance*.

Final, AMOS 24 was used as a statistical tool, and the Bollen-Stine test (93) is employed to test the model fit in this study. The maximum likelihood estimation was used to test the goodness of fit between the data and the model. First, individual-level data (*n* = 365) was analyzed, and the results were described in Table 7. The χ^2^/df of this analysis was 1.694, which reached the standard Goodness of Fit Index (GFI) that was believed within 2. It refers to the proportion of variation and co-variable that the model could explain the observed data. Generally, it is considered that a value higher than 0.9 means that the model has good fitness. Because of the large number of samples and the large degree of freedom in this study, GFI is prone to downward bias, Therefore, GFI has only 0.761 roots mean square error of approximation (RMSEA). The smaller the RMSEA, the better the fit between the hypothesis model and the data. In this study, the RMSEA is 0.093, <0.5 (94). The comparative fit index (CFI) in this study is 0.923, and its value is >0.90 and close to 1, indicating good fitness. Second, team-level data (*N* = 81) was examined. However, the number of team-level samples is too small which may lead to the mismatch between the model and the actual observation data or the model is not ideal. The model was examined by Bootstrapping to generate 1,000 samples. It was found that the measurement model with larger sample size resulted in an insignificant *p*-value of χ^2^ and the other model fitness indexes were in accordance with the criteria (see Table 7). Therefore, it is indicated that the overall measurement model has a reasonable fit.

**Table 7:** Model fit.

**Model fit index**	**Criteria**	**Result 1 (*n* = 365)**	**Result 2 (*N* = 81)**
χ^2^	The small the better	299.790	242.71
χ^2^/df	1 < χ^2^/df < 3	1.694	1.862
GFI	>0.9	0.761	0.870
IFI	>0.9	0.925	0.960
TLI	>0.9	0.909	0.960
CFI	>0.9	0.923	0.960
RMSEA	<0.08	0.093	0.070

### Structural Equation Modeling

#### Path Analysis

**Figure 2:**
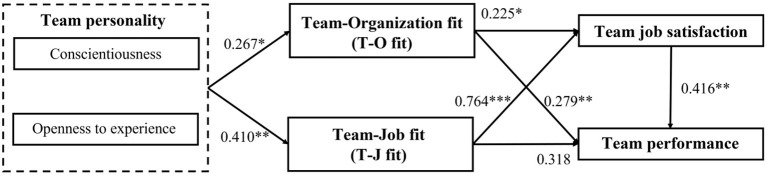
Path analysis of research model (*N* = 81). ^*^p < 0.05; ^**^p < 0.01; ^***^p < 0.001.

The structural equation model is used to examine whether the path between variables is significant, and to verify whether the hypotheses in this study are valid. Based on the above verification results, the measurement model is reasonable, so the following is the result verification of the structural model research hypothesis, the results are shown in Figure 2. Next, Table 8 describes path coefficient and hypothesis testing of theoretical structure model. First, team personality has a significant positive relationship with T-O fit (*t*-value = 2.090, *p* < 0.05) and T-J fit (*t*-value = 2.993, *p* < 0.01). *H1a* and *H1b* are supported, and it indicates that a higher average level of preciseness and openness to experience lead a higher T-O fit and T-J fit. Next, T-O fit and T-J fit have a significant positive relationship with team job satisfaction (*t*-value = 2.292, *p* < 0.05; *t*-value = 5.044, *p* < 0.001). *H2a* and *H2b* are supported, and it indicates that a higher level of T-O fit and T-J fit lead to higher team job satisfaction. Then, T-O fit has a significant positive relationship with team performance (*t*-value = 2.669, *p* < 0.01), but T-J fit does not (*t*-value = 1.954, *p* > 0.05). *H3a* is supported, but *H3b* is not. It indicates that higher T-O fit lead to higher team performance. However, the change in T-J fit has no impact on team performance. Final, team job satisfaction has a significant positive relationship with team performance (*t*-value = 2.671, *p* < 0.01). *H4a* is supported, and it indicates that a higher team job satisfaction leads a higher team performance.

**Table 8:** Path analysis—direct effect.

**Hypothetical path**		**Path Coefficient**		**Standard error**	***t*** **-value**	**Support**
		**Unstandardized**	**Standardized**			
H1a	TP → TO	0.278	0.267	0.133	2.090^*^	Yes
H1b	TP → TJ	0.402	0.410	0.134	2.993^**^	Yes
H2a	TO → SA	0.422	0.225	0.184	2.292^*^	Yes
H2b	TJ → SA	1.522	0.764	0.302	5.044^***^	Yes
H3a	TO → PER	0.296	0.279	0.111	2.669^**^	Yes
H3b	TJ → PER	0.359	0.318	0.184	1.954	NO
H4a	SA → PER	0.236	0.416	0.088	2.671^**^	Yes

* *p < 0.05*,

** *p < 0.01*,

*** *p < 0.001*.

*TP, team personality; TO, T-O fit; TJ, T-J fit; SA, team job satisfaction; PER, team performance*.

#### Indirect Effect Analysis

This section is used to explain whether T-O fit and T-J fit play a mediating role between team personality, team job satisfaction, and team performance. The result shows that T-O fit and T-J fit have a significant mediating effect on team personality and job satisfaction (β = 0.117, *p* < 0.05; β = 0.612, *p* < 0.01). *H5a* and *H6a* are supported. Then, team job satisfaction has a significant mediating effect on T-J fit and team performance (β = 0.359, *p* < 0.05). *H7a* is supported. However, *H5b* and *H6b* have not significant mediating effect (see Table 9).

**Table 9:** Path analysis—indirect effect.

**Hypothetical path**		**Path coefficient (β)**	**Bias-corrected percentile bootstrap confidence intervals (90%)**	**Support**
H5a	TP → TO → SA	0.117^*^	(0.020, 0.310)	Yes
H5b	TP → TO → PER	0.082	(0.015, 0.209)	No
H6a	TP → TJ → SA	0.612^**^	(0.281, 0.995)	Yes
H6b	TP → TJ → PER	0.144	(−0.018, 0.331)	No
H7a	TJ → SA → PER	0.359^*^	(0.134, 0.835)	Yes

* *p < 0.05*,

** *p < 0.01*,

*** *p < 0.001*.

*TP, team personality; TO, T-O fit; TJ, T-J fit; SA, team job satisfaction; PER, team performance*.

## Conclusion

### Discussion

There have been much research studying the P-E theory. They have mainly focused on the relationship between employees and the workplace. Then there are more studies to further explore the impact of P-E fit on performance or job satisfaction. However, there are very few research discussions on the issue of T-E fit. In addition, scholars have advocated and confirmed the importance of team personality [e.g., (9)]. Therefore, the purpose of this study is to understand the mediating effect of T-E fit (T-O fit and T-J fit) between team personality, team job satisfaction and team performance from the perspective of team level.

In this section, this study will further discuss the previous empirical results. First, the research result shows that team personalities (i.e., conscientiousness and openness to experience) positively influence on T-O fit and T-J fit. This result is similar to the findings of previous research [e.g., (21, 60)]. In other words, most members of the team have conscientiousness and openness to experience, and the T-O fit and T-J fit will become stronger. This study further deduces two reasons. First, when members have the high conscientiousness, they can reduce the mistakes in their work. It is especially important for the team. If one member of the team makes a mistake, the work may be affected, and other members need to allocate additional time to solve the problem. Second, when the openness to experience of the team is higher, the team members are willing to brainstorm and think about the problems the team faces, and their acceptance of innovative ideas is also higher.

Second, there are many researches discussing the correlation between P-E fit (organization and job), and job satisfaction and performance. They found that P-E fit (organization and job) significantly and positively affects job satisfaction [e.g., (2, 6, 65, 66)] or performance [e.g., (52)]; satisfaction is also positively related to performance [e.g., (49–51)]. However, few studies have examined the team level [e.g., (71)]. This study empirically demonstrated the relationship between T-O fit, T-J fit, team job satisfaction, and team performance. The results showed that T-O fit significantly and positively influenced team job satisfaction and team performance; T-J fit was significantly and positively related to team job satisfaction, but not team performance; team job satisfaction also positively influenced team performance. Apparently, the results on the relationship between the environment fit, job satisfaction, and performance were similar at the team level and at the individual level. The more consistent the values of team members are with the organization; the more team satisfaction and performance can be achieved. This verified result is similar to the proposition proposed by scholars (68). Teams that have a good relationship or shared values with the organization will also perform well. The more the competencies of most team members can meet the job requirements, the higher the team's job satisfaction will increase. In addition, when team job satisfaction rises, it leads to an increase in performance. However, the effect of T-J fit on team performance was not significant. This result is different from previous researches [e.g., (65)]. The inference may be due to the reason that this study discussed the team-level and multiple industries, whereas previous researches explored the individual-level and single industry. Further, the largest number of respondents and teams interviewed in this study were in the information department and information services industry. Information personnel are in a support role and often have to face and solve complex problems but their performance is difficult to measure.

Third, the results of this study showed that both T-O fit and T-J fit had a significant positive mediating effect between team personality and team job satisfaction; team job satisfaction had a significant positive mediating effect between T-J fit and team performance. Peeters et al. (22) had found the team personality and proposed that conscientiousness positively affects team performance. Furthermore, Lim et al. (95) had pointed out that openness to experience is related to team adaptability. When a team has better personality than other teams in adapting to the changing environment, which improve T-J fit, reduce the sense of incompetence, and greatly improve job satisfaction. The more the employees' professional skills meet the job requirements, the higher the sense of accomplishment they get at work, which generate more satisfaction with the work and ultimately improve team performance. On the other hand, T-O fit has a good predictability for the team members' behaviors. By selecting the employees who fit with the organization, it is conducive to enhance the communication and cooperation among the members of the organization, increase the cohesion and efficiency of the organization, and improve job satisfaction and performance. However, to a certain extent, it may lead to organizational rigidity and conservatism, lack of innovation, and reduce organizational adaptability. As a result, teams and organizations should be flexible in order to contribute to performance growth. Moreover, Khadivi et al. (70) concluded that job satisfaction affects organizational performance and that job satisfaction is influenced by other factors. The results of this study are consistent with their arguments. Apparently, team job satisfaction not only positively affects performance, but it also plays a mediator between T-J fit and team performance. In other words, T-J fit needs to be influenced by team job satisfaction to affect team performance. If team members are competent in team work, team job satisfaction will rise and team performance will be further increased.

In conclusion, the results of this study confirm that team personality is an effective predictor, which can be used to select team members and configure tasks. T-O fit and T-J fit can not only predict team job satisfaction but also contribute to the development of team norms and influence the effectiveness of behavior at the team-level. Since there are a lot of researches on the role of personal characteristics in the context of the collaborative office, there are few researches on the role of T-E fit in a team. Since many current researches focus on the impact of individual-level personality traits and environmental fit on satisfaction and performance. Relatively few researches have explored the team-level personality. However, some scholars have concerned about team-level issues and argued that individual-level and team-level personalities are different (27). Hence, this study promotes an in-depth understanding of the interaction between these team-level phenomena, which is also beneficial to theory and practice.

This study investigates the team members in the enterprise and discusses the mediating effect of team and environment (organization and job) fit on team personality, team job satisfaction and team performance, and provides a certain empirical and theoretical basis on how to improve the fit. The main contributions of this study are as follows. First, discussing team personality. Through the study of the relationship between team personality combination and team performance and team job satisfaction, analyze the influence of different personality combinations on team performance. Taking the team member personality combination as the starting point, explore the team combination that is conducive to team performance and team job satisfaction. Provide powerful help for the company in the construction of the team, so that the recruited object not only meets the needs of the job position, but also considers the complementary relationship between the existing members of the team and the new members, and meets the fit between people, job, and organizations. Second, exploring T-O fit and T-J fit. The research findings on T-O fit and T-J fit have very important theoretical and practical implications. In terms of theory, the P-O fit and P-J fit in the P-E fit theory have been extended to the team level. Additionally, this study not only verified the predictability of T-O fit and T-J fit on team job satisfaction and team performance, but also explored their mediating roles in team personality, job satisfaction, and team performance. In terms of practice, the findings of this study provide a new recruitment model for enterprises to attract and retain key employees, theoretical support for personnel recruitment research, and a reference for organizational culture research. Moreover, the recruitment, assessment, and cultivation of talents not only consider whether the individual's abilities are consistent with their job (T-J fit) but more importantly, use effective methods to measure the relationship between their individual characteristics and organizational characteristics compatibility. Therefore, the research on T-O fit provides favorable support for human resource management, highlighting a new type of management concept and development strategy.

### Management Implications

The purpose of this study is to explore the relationship among team personality, T-O fit and T-J fit, team job satisfaction, and team performance. The mediating effect of T-O fit and T-J fit on the relationship between team personality, team job satisfaction, and team performance was investigated. Then, this study found that T-O fit and T-J fit are enhanced to improve team job satisfaction and team performance. Thus, the management implications are further discussed from the following perspectives. First, personnel recruitment and selection. When recruiting new employees, the organization should strengthen the test of the personal values of job applicants and select employees with a high conformity with the organization's values, which help improve their job satisfaction and increase team performance. Second, organizational socialization. T-O fit and T-J fit are closely related to employees' attitudes and behaviors. Hence, in the socialization process, organizations should arrange training not only on job content and skills, but also on organizational culture to increase the value fit between the organization and employees. Employees can not only improve their work efficiency but also strengthen their sense of identity with the organization. Then, the morale and stability of the team also increase. Third, human resource management. Managers can use various measures such as regular meetings to continuously achieve value recognition with employees. In the performance management indicators, the value compatibility should also be regarded as an important indicator. Final, career development. Employees are able to continuously assess their T-O fit and T-J fit to help plan their careers. Through these assessments, employees can understand whether they are suitable for their current positions and teams. On the other hand, the organization understands T-O fit and T-J fit of employees to adjust and propose appropriate HR strategies. The more flexible an organization is, the more it can respond to changes in the external environment.

### Limitations and Future Research

Given the limited capacity, resources and time, there are still some inadequacies in this study. There are some limitations in this study, which can remind us to pay attention to the future research direction. First, the survey results of the scale in this study are self-reports from employees. This method is often criticized for causing common method variance (CMV). Therefore, this study adopts some preventive measures to reduce errors and avoid unnecessary interference to answerers, such as using more rigorous procedures to construct the scale, and carefully consider the text. In addition, this study refers to the suggestions of Podsakoff et al. (96) and uses an anonymous questionnaire. However, whether the respondents fill in the questionnaire truthfully cannot be guaranteed. Future research should focus on more objective behavioral measurements, such as using actual data (e.g., salary increase percentage, team turnover) to evaluate performances [e.g., (97)]. Final, this study selected two factors (conscientiousness and openness to experience) from the Big Five personality traits based on previous literature reviews. However, scholars have different definitions of personality traits. Not all personality traits can be transformed into team personality. Future research can refer to the personality traits and team personality proposed by different scholars to further explain the team personality more clearly and make the research more complete.

## Data Availability Statement

The raw data supporting the conclusions of this article will be made available by the authors, without undue reservation.

## Author Contributions

Conceptualization: Y-TL, Y-CL, and S-CC. Methodology: OS, UR, AR, and S-CC. Validation: T-HC, Y-CL, and AR. Formal analysis: XL, UR, and Y-TL. Investigation: XL and Y-TL. Writing—original draft preparation: XL, Y-TL, Y-CL, and S-CC. Writing—review and editing: OS, T-HC, UR, and AR. Visualization: Y-TL and Y-CL. All authors have read and agreed to the published version of the manuscript.

## Conflict of Interest

The authors declare that the research was conducted in the absence of any commercial or financial relationships that could be construed as a potential conflict of interest.

## Publisher's Note

All claims expressed in this article are solely those of the authors and do not necessarily represent those of their affiliated organizations, or those of the publisher, the editors and the reviewers. Any product that may be evaluated in this article, or claim that may be made by its manufacturer, is not guaranteed or endorsed by the publisher.
